# Evaluation of the Bishop Score in Comparison With Ultrasonographic Markers in Predicting Successful Induction of Labor

**DOI:** 10.7759/cureus.103831

**Published:** 2026-02-18

**Authors:** Antonios Michail, Ekaterini Domali, George Daskalakis, Panagiotis Antsaklis

**Affiliations:** 1 1st Department of Obstetrics and Gynecology, National and Kapodistrian University of Athens, Alexandra General Hospital, Athens, GRC

**Keywords:** bishop score, cesarean section, cesarean section (cs), induction of labor, ultrasound cervical assessment, vaginal delivery prediction

## Abstract

Background

Induction of labor (IOL) is a common obstetric intervention, yet accurately predicting successful vaginal delivery remains challenging. The Bishop score is widely used to assess cervical readiness, although its predictive performance is limited by subjectivity and variability. Recent studies suggest that ultrasound-based or composite scoring systems may improve the prediction of induction outcomes. The objective of this study was to compare, within a prospective cohort of women undergoing labor induction at ≥34 weeks of gestation, the predictive performance of the Bishop score and a novel ultrasound-based composite induction score for the primary outcome of vaginal delivery.

Methods

This prospective observational cohort study included 200 women undergoing IOL at ≥34 weeks of gestation for standard obstetric indications. Prior to induction, all participants underwent digital cervical examination for calculation of the Bishop score and standardized ultrasound assessment. The novel induction score integrated sonographic parameters, including cervical length, fetal head characteristics, cervical elastography, and cervical angulation. The primary outcome was vaginal delivery. Receiver operating characteristic curve analysis was used to assess predictive performance.

Results

The mean maternal age was 30.8 ± 5.4 years, and 56.0% of participants were nulliparous. Vaginal delivery occurred in the majority of cases. The Bishop score demonstrated good predictive ability for vaginal delivery (AUC 0.784, 95% CI 0.686-0.882; p < 0.001). The novel induction score showed significantly higher discriminative performance (AUC 0.891, 95% CI 0.827-0.955; p < 0.001). Clinically relevant cutoffs of the new score provided a more favorable balance between sensitivity and specificity compared with the Bishop score.

Conclusions

While the Bishop score remains a useful baseline tool, the novel ultrasound-based induction score demonstrated superior predictive accuracy for vaginal delivery. These findings suggest that incorporating objective sonographic parameters into pre-induction assessment may improve risk stratification and support individualized clinical decision-making. External validation is warranted before routine clinical implementation.

## Introduction

Induction of labor (IOL) constitutes one of the most frequently performed obstetric interventions worldwide, with steadily increasing rates over recent decades. This trend reflects shifts in obstetric practice driven by advancing maternal age, the growing prevalence of medical comorbidities, and a higher proportion of post-term and high-risk pregnancies. Despite its widespread use, IOL remains clinically challenging, as failure to achieve vaginal delivery is associated with prolonged labor, increased cesarean section rates, and elevated maternal and neonatal morbidity [1,2].

Accurate assessment of cervical readiness is central to the prediction of induction success. The Bishop Score, first described in 1964, remains the most commonly applied clinical tool for this purpose. By integrating five digitally assessed components-cervical dilatation, effacement, consistency, position, and fetal station-the Bishop score provides a composite estimate of cervical favorability [3]. Although extensively validated and deeply embedded in clinical practice, its predictive performance has been shown to vary considerably, particularly in nulliparous women and in cases with intermediate scores. Moreover, its reliance on subjective digital examination introduces significant inter- and intra-observer variability, limiting its reproducibility and clinical precision [4,5].

In response to these limitations, obstetric ultrasound has gained increasing attention as an objective modality for assessing cervical and fetal head characteristics prior to labor induction. Parameters such as cervical length, anterior cervical angle, fetal head-perineum distance, head position, and cervical elastography provide quantitative, reproducible information on the biomechanical and anatomical readiness for labor. Multiple studies have demonstrated that ultrasound-derived measurements may equal or surpass the predictive value of the Bishop score, particularly when used in combination, offering improved discrimination for successful vaginal delivery following induction [6-8]. More recently, composite ultrasound-based indices have been proposed as integrated predictors, aiming to capture the multifactorial nature of labor progression more effectively than isolated parameters [9,10].

Nevertheless, despite accumulating evidence supporting ultrasound-based assessment, its optimal role in routine clinical decision-making remains uncertain. Considerable heterogeneity persists across studies regarding the choice of parameters, scoring systems, and proposed thresholds, and there is no consensus as to whether ultrasound-derived indices should complement or potentially replace traditional clinical scoring systems. Against this background, a systematic comparative evaluation of the Bishop score and ultrasound-based predictors is warranted in order to clarify their relative and combined predictive utility for successful IOL.

The aim of this prospective cohort study was to evaluate, within a defined population of women undergoing IOL, the comparative predictive performance of the Bishop score and a novel ultrasound-derived composite score for successful vaginal delivery, without implying external generalizability beyond the present cohort.

## Materials and methods

This was a prospective observational cohort study conducted at Alexandra General Hospital, 1st Department of Obstetrics and Gynecology, National and Kapodistrian University of Athens, a tertiary referral center. Women admitted for IOL between October 1, 2023 and January 1, 2026 were consecutively screened for eligibility to minimize selection bias and ensure representativeness of the study population.

Eligible participants were women aged ≥18 years with a singleton pregnancy, cephalic presentation, and gestational age ≥34 weeks, scheduled for IOL for accepted medical or obstetric indications, including post-term pregnancy, hypertensive disorders of pregnancy, diabetes mellitus, or other guideline-based indications. Exclusion criteria comprised multiple gestation, non-cephalic fetal presentation, placenta previa, history of uterine rupture, or any absolute contraindication to vaginal delivery. Women who did not meet the inclusion criteria or declined participation were excluded from further analysis.

No participants were excluded after enrollment; women who developed intrapartum complications after initiation of induction remained in the analysis, and outcomes were classified based on the final mode of delivery.

Because this was a prognostic accuracy study comparing two prediction tools measured in the same cohort, no separate control group was defined.

Clinical assessment

Prior to the initiation of labor induction, all participants underwent a standardized digital cervical examination performed by experienced obstetricians. Cervical status was assessed using the Bishop score, calculated in accordance with the original scoring system. To reduce inter-observer variability, examinations were conducted by clinicians routinely involved in intrapartum care and familiar with standardized cervical assessment. Labor induction was carried out according to institutional protocols, in line with current national and international recommendations, with the choice of induction method determined by clinical indication and cervical favorability.

Ultrasound assessment

Ultrasound evaluation was performed prior to induction by trained operators who were blinded to the results of the clinical cervical examination. All ultrasound examinations were performed by two senior obstetricians with formal certification in obstetric ultrasonography and more than eight years of clinical experience in third-trimester and intrapartum ultrasound. Each operator performs approximately 800-1,000 obstetric ultrasound examinations annually. Both operators were specifically experienced in transvaginal cervical length measurement and fetal head position assessment. A standardized measurement protocol was used for all examinations, and the operators were blinded to the Bishop score findings at the time of ultrasound evaluation.

Transvaginal ultrasound was used to assess the cervical length and anterior cervical angle, while transperineal ultrasound was employed to evaluate fetal head-perineum distance and fetal head position. Cervical elastography was additionally performed to provide information on cervical tissue stiffness.

To enhance predictive accuracy, selected ultrasound parameters were integrated into a composite ultrasound-based score, developed on the basis of previously reported associations and internal modelling. More specifically, a composite ultrasound-based score was constructed to quantify cervical and fetal head readiness for labor induction by integrating five objective sonographic parameters. Each parameter was scored on a three-point scale (0-2), with higher scores indicating more favorable conditions for successful induction. The total score ranged from 0 to 10.

Cervical length measured by transvaginal ultrasound was scored as 0 for values >20 mm, 1 for 11-20 mm, and 2 for <11 mm. Fetal head position assessed by transperineal ultrasound was scored as 0 for occiput posterior (OP), 1 for occiput transverse (OT), and 2 for occiput anterior (OA). The head-perineum distance was scored as 0 when >50 mm, 1 when 40-50 mm, and 2 when <40 mm. Cervical elastography values were obtained separately for the inner, middle, and outer thirds of the cervix; a score of 0 was assigned when stiffness exceeded 10 kPa, 8 kPa, and 6 kPa respectively, a score of 1 for intermediate values (8.1-10 kPa, 6.6-8 kPa, and 5.1-6 kPa), and a score of 2 when stiffness values were below 8.1 kPa, 6.6 kPa, and 5.1 kPa, respectively. Cervical angulation was evaluated using the anterior angle (AA), posterior angle (PA), and their sum (AA+PA). Scores of 0, 1, and 2 were assigned for AA <90°, 90°-104°, and >104°, respectively; for PA <90°, 90°-99°, and >99°; and for AA+PA <170°, 170°-190°, and >190°, respectively. The mean stiffness value across the inner, middle, and outer cervical thirds was used to assign the final cervical elastography score.

The composite ultrasound-based induction score was developed by the investigators based on ultrasound parameters that have been consistently evaluated in previously published studies assessing the prediction of successful induction of labor. The selection of parameters was informed by evidence demonstrating the predictive value of transvaginally measured cervical length [5-7,9,10], transperineal ultrasound assessment of fetal head position and head-perineum distance as objective markers of fetal head engagement [11-13], cervical angulation and uterocervical angle measurements reflecting cervical-uterine axis alignment [14], and cervical tissue stiffness assessed by cervical elastography as a biomechanical indicator of cervical ripening [15,16].

Although each of these ultrasound parameters has been previously studied individually or in combination, the specific integration of these variables into a single composite score, including the categorization, scoring thresholds, and total score range used in the present study, has not been previously published and was developed de novo for research purposes.

Based on the composite ultrasound score, 58 women (29.0%) were classified as low probability (score 0-3), 96 women (48.0%) as intermediate probability (score 4-6), and 46 women (23.0%) as high probability (score 7-10). Ultrasound findings and the composite score were recorded for study purposes and did not determine the choice of induction method. All ultrasound measurements were obtained using standardized techniques, and repeat assessments were performed when necessary to ensure image quality and measurement reliability.

Outcome definition

The primary outcome of the study was successful induction of labor, defined as achievement of vaginal delivery following induction. Unsuccessful induction was defined as cesarean delivery performed for failed induction or arrest of labor progress. Outcome classification was based on delivery records and operative reports, ensuring objective and reproducible endpoint assessment.

Statistical analysis

Statistical analysis was performed using IBM SPSS Statistics for Windows, Version 26 (Released 2018; IBM Corp., Armonk, New York, United States). Continuous variables were assessed for normality using the Shapiro-Wilk test and visual inspection of histograms. Normally distributed continuous variables are presented as mean ± standard deviation (SD), whereas non-normally distributed variables are expressed as median (interquartile range). Categorical variables are presented as frequencies and percentages.

The predictive performance of the Bishop score and the novel induction score for vaginal delivery was evaluated using receiver operating characteristic (ROC) curve analysis. Discrimination was quantified by the area under the curve (AUC) with corresponding 95% confidence intervals (CIs). ROC curves were constructed under the nonparametric assumption, and statistical significance of each AUC was assessed against the null hypothesis of an area equal to 0.5. Sensitivity and specificity were calculated for selected cutoff values derived from ROC coordinates, emphasizing clinically relevant thresholds.

All statistical tests were two-sided, and a p-value < 0.05 was considered statistically significant.

The individual ultrasound parameters included in this composite score have been previously described in the literature as predictors of labor induction outcome. However, the categorical scoring system and the integration of parameters into a composite ultrasound score were developed for the purposes of this study. Predictive (criterion) validity of the composite score was assessed internally using ROC curve analysis and AUC estimation. External validation in independent cohorts was not performed and should be addressed in future studies.

## Results

A total of 200 women were included in the study. The mean maternal age was 30.8 ± 5.4 years. More than half of the participants were nulliparous (56.0%), while 44.0% were multiparous.

Regarding indications for labor induction, post-term pregnancy was the most common reason, accounting for 62 women (31.0%), followed by hypertensive disorders of pregnancy in 46 cases (23.0%) and premature rupture of membranes in 38 cases (19.0%). Fetal growth restriction was observed in 24 women (12.0%), whereas other maternal or fetal indications accounted for 30 inductions (15.0%).

Most inductions were performed at a gestational age greater than 36 weeks (148/200, 74.0%), while 52 women (26.0%) were induced between 34 and 36 weeks of gestation. With respect to the induction method, dinoprostone was used in 100 cases (50.0%), oxytocin alone in 30 cases (15.0%), and a combined regimen of dinoprostone and oxytocin in 70 cases (35.0%).

Table 1 summarizes the demographic and obstetric characteristics of the 200 women included in the study.

**Table 1:** Demographic and obstetric characteristics of the study population. SD: standard deviation. The data has been represented as N, %, Mean±SD. The level of significance was set as p<0.05.

Variable	Total (N = 200)
Age (years), mean ± SD	30.8 ± 5.4
Parity	
Nulliparous, n (%)	112 (56.0)
Multiparous, n (%)	88 (44.0)
Indication for induction	
Post-term pregnancy, n (%)	62 (31.0)
Hypertensive disorders of pregnancy, n (%)	46 (23.0)
Premature rupture of membranes, n (%)	38 (19.0)
Fetal growth restriction, n (%)	24 (12.0)
Other indications, n (%)	30 (15.0)
Gestational age at induction	
34–36 weeks, n (%)	52 (26.0)
>36 weeks, n (%)	148 (74.0)
Induction medicine	
Dinoprostone, n (%)	100 (50.0)
Oxytocin, n (%)	30 (15.0)
Dinoprostone + Oxytocin, n (%)	70 (35.0)

Predictive performance of the Bishop score for vaginal delivery

**Figure 1:**
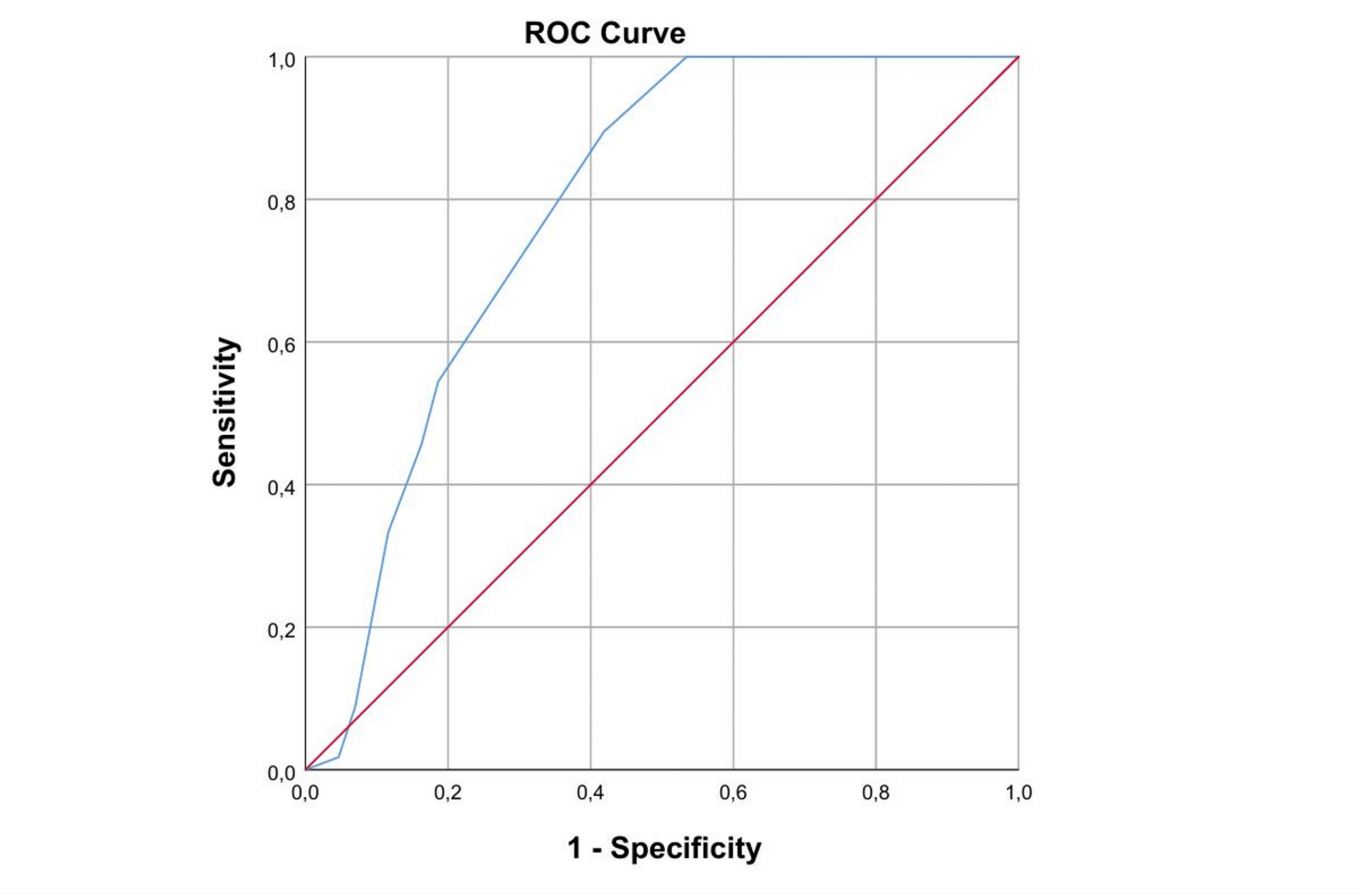
ROC curve analysis for the predictive role of the Bishop score. The level of statistical significance was set as p<0.05.

ROC curve analysis demonstrated that the Bishop score was a significant predictor of vaginal delivery (Figure 1).

The area under the ROC curve (AUC) was 0.784 (standard error 0.050), indicating good discriminative ability. The AUC was significantly greater than the null value of 0.5 (p < 0.001), with an asymptotic 95% CI ranging from 0.686 to 0.882.

Evaluation of the ROC coordinates showed that lower Bishop score thresholds were associated with very high sensitivity but low specificity, whereas increasing cutoff values resulted in a progressive trade-off between sensitivity and specificity. Specifically, a Bishop score cutoff of ≥8.5 yielded a sensitivity of 89.5% with a false-positive rate of 41.9%, whereas a cutoff of ≥9.25 reduced sensitivity to 54.4% while improving specificity (1 − specificity = 18.6%). At higher thresholds (≥11.5), specificity was high but sensitivity dropped markedly (8.8%).

Overall, these findings indicate that the Bishop score has clinically meaningful accuracy for predicting vaginal delivery, performing best as a rule-out tool at lower cutoffs to identify women with a high likelihood of successful vaginal birth, while higher cutoffs may be useful when prioritizing specificity.

Predictive performance of the new induction score for vaginal delivery

**Figure 2:**
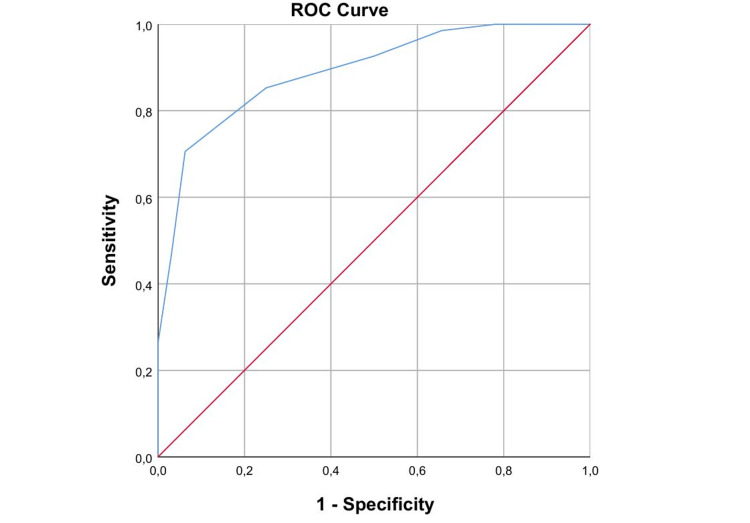
ROC curve analysis for the predictive role of the new score. The level of statistical significance was set as p<0.05.

ROC curve analysis demonstrated that the new induction score showed excellent discriminative ability for predicting vaginal delivery (Figure 2).

The AUC was 0.891 (standard error 0.033), which was significantly greater than 0.5 (p < 0.001), with a 95% CI of 0.827 to 0.955.

Analysis of the ROC coordinates indicated that lower cutoff values were associated with very high sensitivity, while progressively higher thresholds improved specificity. A cutoff value of ≥3.5 achieved a sensitivity of 85.3% with a false-positive rate of 25.0%, whereas a cutoff of ≥4.5 provided a more balanced trade-off, yielding a sensitivity of 70.6% and a false-positive rate of only 6.3%. At higher thresholds (≥6.5), specificity reached 100%, but sensitivity was substantially reduced (26.5%).

Overall, the new score demonstrated superior predictive accuracy compared with the Bishop score, indicating a stronger ability to discriminate between women who would and would not achieve vaginal delivery, particularly at clinically relevant cutoff values.

Stratified analysis by parity and maternal age

**Figure 3:**
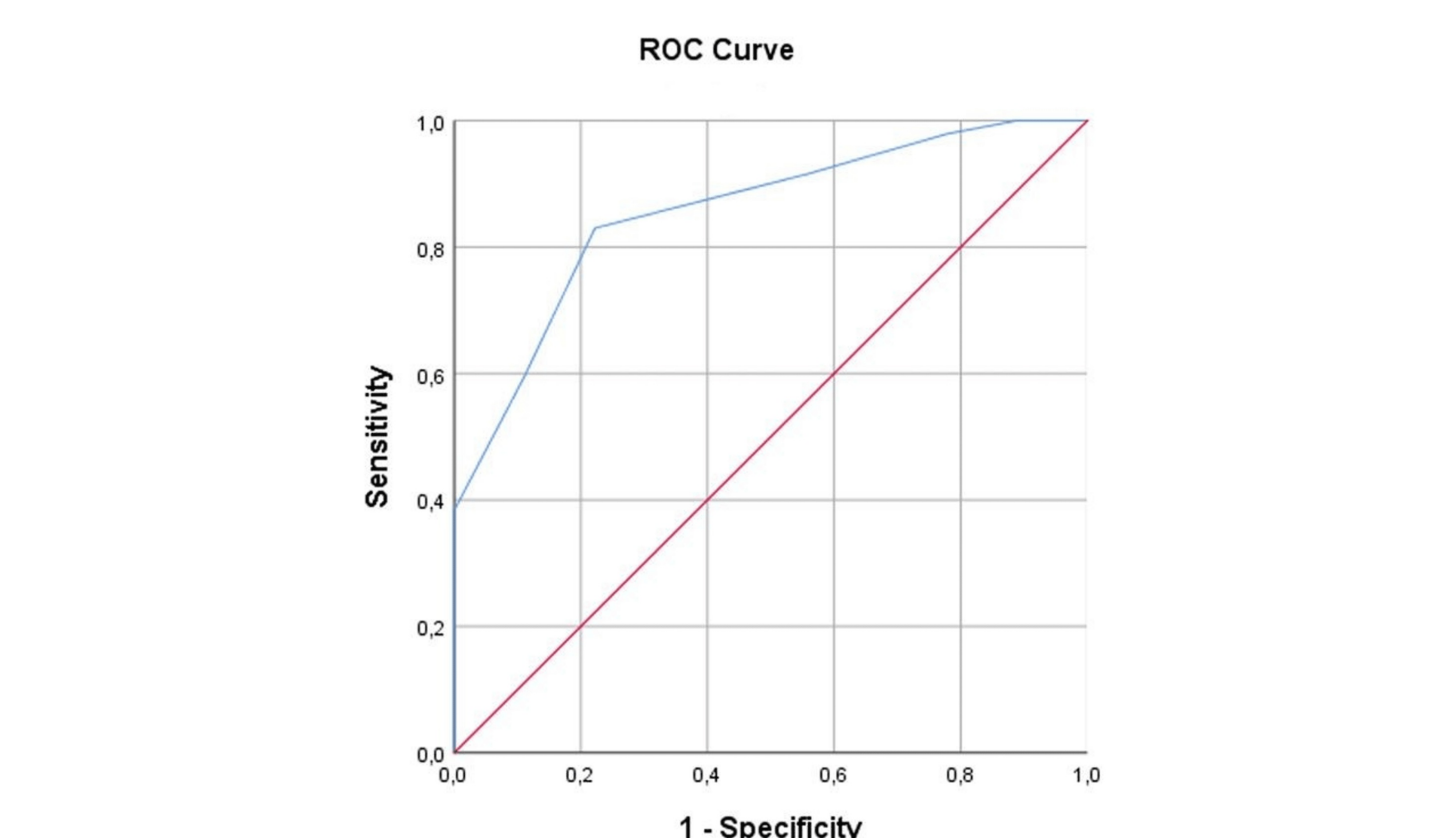
ROC curve of the composite ultrasound score for prediction of vaginal delivery among nulliparous women.

Stratified ROC curve analysis demonstrated consistently high discriminative performance of the composite ultrasound score across parity subgroups. Among nulliparous women, the AUC was 0.856 (95% CI 0.735-0.977, p = 0.001), indicating excellent predictive accuracy for vaginal delivery (Figure 3).

**Figure 4:**
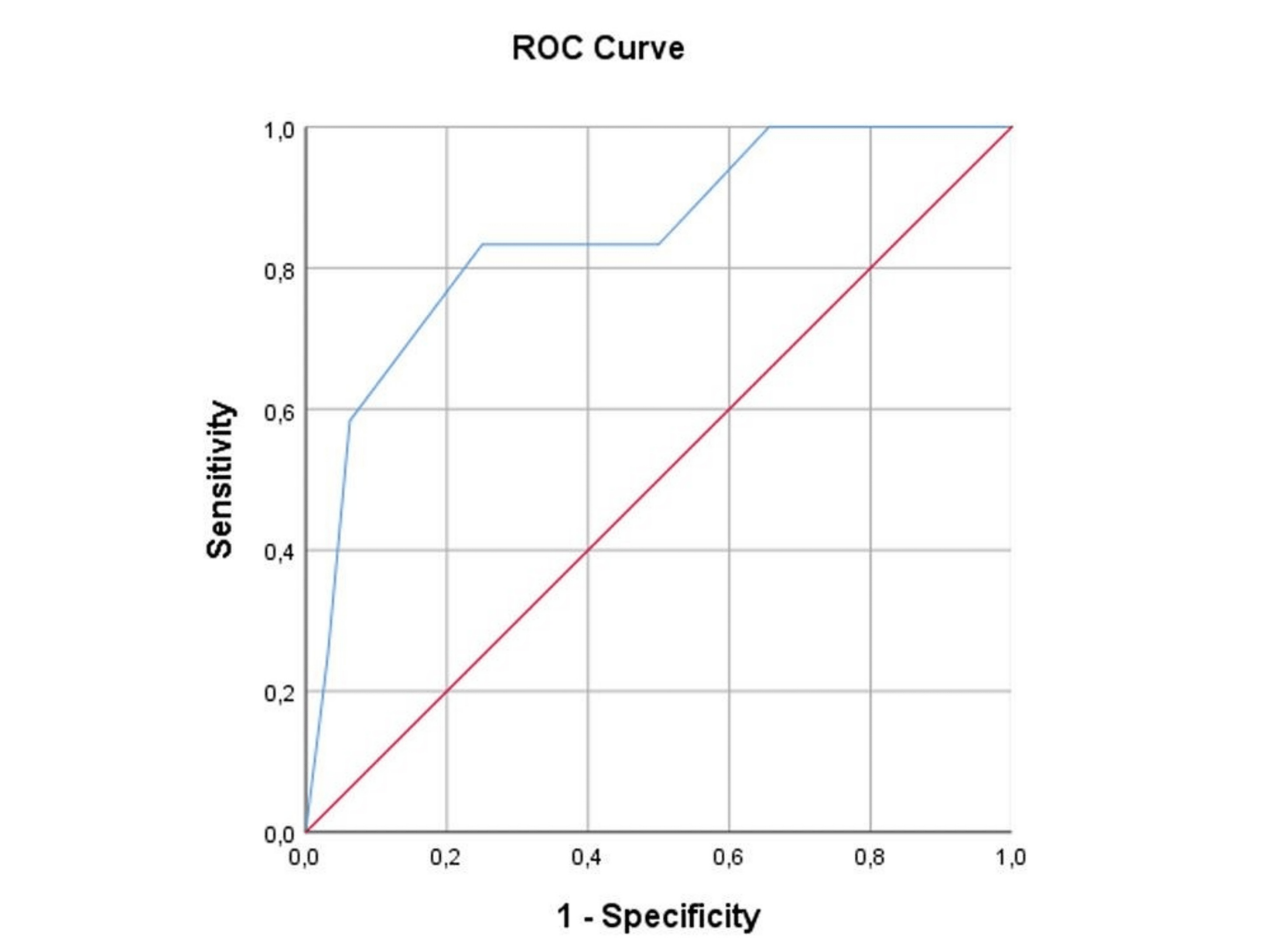
ROC curve of the composite ultrasound score for prediction of vaginal delivery among multiparous women.

Similarly, in multiparous women, the AUC was 0.845 (95% CI 0.712-0.978, p < 0.001), also reflecting strong discrimination (Figure 4).

The overlapping CIs between parity groups suggest that the predictive performance of the composite score does not differ meaningfully between nulliparous and multiparous women. These findings indicate that parity does not substantially modify the discriminative ability of the proposed model.

In multivariable logistic regression analysis adjusting for maternal age, the composite ultrasound score remained independently associated with vaginal delivery (adjusted OR 2.54, 95% CI 1.32-4.88, p = 0.005). Maternal age was not independently associated with induction success (adjusted OR 0.91 per year increase, 95% CI 0.80-1.05, p = 0.198).

## Discussion

In this study, we assessed the predictive ability of the Bishop score and a new induction score for vaginal delivery following labor induction. The Bishop score demonstrated good discrimination, while the new score showed excellent performance, indicating a meaningful improvement in prediction. These findings support the concept that while digital cervical assessment remains clinically useful, enhanced scoring approaches may better stratify the likelihood of successful vaginal birth after induction.

The Bishop score continues to be embedded in modern clinical decision-making, including guideline frameworks that classify cervical status as unfavorable vs. favorable using cutoffs around 6-7 to guide choice of cervical ripening versus direct induction methods. For example, contemporary guidance explicitly uses Bishop thresholds to support method selection (e.g., pharmacologic vs. mechanical ripening or oxytocin with amniotomy when favorable) [17-19]. Nevertheless, the evidence base consistently recognizes that the Bishop score is constrained by interobserver variability and by the limited objectivity of several components, which may weaken predictive precision in real-world settings [20]. In this context, our observed AUC (~0.78) is consistent with the “moderate-to-good” range commonly reported across diverse settings and induction indications, but also highlights why the Bishop score alone may not fully capture the complex determinants of induction success.

Recent literature increasingly emphasizes that alternative approaches, particularly those incorporating ultrasound markers or multivariable/computational models, can outperform the Bishop score. Multiple contemporary studies comparing digital Bishop assessment to transvaginal sonographic parameters (especially cervical length, and sometimes additional ultrasound features) suggest that ultrasound may offer equal or improved prediction, although results vary by population, endpoint definitions, and induction protocols [21-24]. Importantly, these studies also reinforce a key limitation of Bishop scoring: variability between examiners and settings can introduce measurement noise that reduces predictive discrimination [21]. The strong performance of our score is therefore aligned with the current direction of the field: moving from purely subjective cervical examination toward more standardized or data-driven prediction.

Beyond ultrasound, recent work using machine learning and computational modeling has shown particularly promising discriminative performance for predicting vaginal delivery after induction. A 2024 study developing computational learning models for vaginal delivery after induction reported strong AUROC performance in validation workflows [25]. Similarly, a 2023 machine-learning-based approach that compared ultrasound and Bishop-derived assessment frameworks highlighted how algorithmic models can increase predictive accuracy and potentially support more individualized induction planning [26]. Of particular relevance, a 2025 report directly framed the Bishop score’s limitations and described superior discrimination for a machine-learning model compared with Bishop scoring in predicting induction success, consistent with the direction suggested by our findings, namely, that improved scoring approaches can yield clinically meaningful gains [27]. Collectively, these contemporary data support the plausibility that a more informative scoring system can meaningfully improve prediction over Bishop alone, particularly when applied at clinically actionable thresholds.

Clinically, the implications are tangible. Accurate pre-induction prediction supports shared decision-making and helps tailor the induction strategy (e.g., selecting cervical ripening approaches, anticipating duration and resource utilization, and counseling about cesarean likelihood). In many guideline settings, the Bishop score already influences the selection of ripening and induction methods [17-19]. A higher-performing score such as ours may support risk stratification, inform counselling, unnecessary escalation, and patient dissatisfaction. From the ROC coordinates in our data, the new score provided favorable trade-offs at clinically relevant cutoffs (e.g., thresholds around 3.5-4.5), suggesting that it may be particularly useful in identifying women with a high probability of vaginal delivery while maintaining acceptable false-positive rates. If validated externally, such a tool could be integrated into standardized induction pathways to support consistent decision-making across clinicians and shifts.

Several limitations should be considered. First, the analysis appears to be based on a single dataset without external validation; therefore, generalizability to other settings, case-mixes, and induction protocols remains uncertain. Second, the ROC output indicates ties between positive and negative outcome groups, a common issue with discrete clinical scores, which can introduce bias in nonparametric ROC estimates and should be acknowledged when interpreting precision (as flagged by the statistical output). Third, we focused on vaginal delivery as the primary endpoint; additional clinically important outcomes, such as induction-to-delivery time, uterine tachysystole, postpartum hemorrhage, neonatal acid-base status, NICU admission, and indications for cesarean, were not incorporated into the predictive framework. Fourth, performance may vary by parity, gestational age strata (e.g., 34-36 vs. >36 weeks), and induction indication; stratified analyses and interaction testing would strengthen interpretability and clinical translation. Moreover, as with all prediction tools, a high AUC does not automatically translate to net clinical benefit; decision-curve analysis and implementation studies would help define whether using our new score changes management in ways that improve outcomes.

Despite the encouraging findings, the reproducibility of the proposed method warrants further investigation. Although standardized ultrasound measurement protocols were applied, formal quantitative assessment of intra- and interobserver agreement was not performed. Given that certain components of the composite score may be influenced by operator experience, systematic evaluation of methodological reliability represents an essential step prior to broader clinical implementation. In addition, the present study was conducted within a single clinical setting and specific patient population, which may limit the immediate generalizability of the findings. External validation in independent cohorts with varying demographic and obstetric characteristics is necessary to confirm the stability and transportability of the model across different clinical environments. Finally, while this study focused on predicting vaginal delivery, the true clinical value of any predictive tool lies in its impact on decision-making and maternal and neonatal outcomes. Future studies should therefore assess whether integration of the composite score into routine clinical practice improves counseling accuracy, reduces unnecessary cesarean deliveries, and contributes to optimized perinatal outcomes. Such analyses would allow evaluation not only of predictive discrimination but also of real-world clinical effectiveness.

## Conclusions

In conclusion, our findings confirm that the Bishop score remains a clinically useful baseline predictor aligned with current guideline-based practice. The proposed composite ultrasound score demonstrated superior discriminative performance for predicting vaginal delivery within this cohort, suggesting that the integration of sonographic parameters may enhance pre-induction risk stratification. However, the model should be considered exploratory. It has not undergone external validation, and operator-dependent reproducibility was not formally quantified. Accordingly, independent validation studies, assessment of inter- and intra-observer reliability, and evaluation of model calibration are essential before routine clinical implementation can be considered. Future investigations should also explore performance across clinically relevant subgroups, including parity, prior obstetric history, and maternal age, to assess generalizability and potential effect modification.

Importantly, while this study focused on predicting vaginal delivery, the ultimate goal of labor induction is the optimization of both maternal and neonatal outcomes. Future research should therefore incorporate perinatal outcome measures, including neonatal condition at birth (Apgar scores, acid-base status), need for resuscitation, admission to neonatal intensive care, and early neonatal course, in order to evaluate not only predictive discrimination but also the broader clinical impact and perinatal relevance of the model. Taken together, these findings provide a foundation for further refinement and validation of ultrasound-based prediction tools aimed at improving individualized induction counseling while ensuring maternal and neonatal safety.
